# Supplementary material for: MiR-15b-5p Expression in the Peripheral Blood: A Potential Diagnostic Biomarker of Autism Spectrum Disorder
Source: Brain Sci. 2022 Dec 22;13(1):27. doi: 10.3390/brainsci13010027 (PMC9855964; doi:10.3390/brainsci13010027)
Supplement: Supplementary file 1 [file brainsci-13-00027-s001.zip › brainsci-2105386-supplementary.pdf]

Table S1. Primers for the qPCR experiment.

miRNAs	Primer sequence (5′ - 3′)
hsa-miR-15b-5p	TAGCAGCACATCATGGTTTACA
hsa-miR-15a-5p	AGCAGCACATAATGGTTTGTGAAA
hsa-miR-19b-3p	TGTGCAAATCCATGCAAAACTGAAA
hsa-miR-27a-3p	TTCACAGTGGCTAAGTTCCGC
hsa-miR-106b-5p	TAAAGTGCTGACAGTGCAGAT
hsa-miR-320a-5p	CTTCTCTTCCCGGTTCTTCCAAA
hsa-miR-320a-3p	AAAAGCTGGGTTGAGAGGGCGA
hsa-miR-451a	AAACCGTTACCATTACTGAGTT
hsa-miR-494-5p	AGGTTGTCCGTGTTGTCTTCTCTAA
hsa-miR-494-3p	TGAAACATACACGGGAAACCTCAAA

miRNA/miR, microRNA; hsa : Homo Sapiens.

Table S2. Predicted target genes of miR-15b-5p using miRDB.

Target Rank	Target Score	miRNA Name	Gene Symbol
1	100	hsa-miR-15b-5p	PAPPA
2	100	hsa-miR-15b-5p	FASN
3	100	hsa-miR-15b-5p	UNC80
4	100	hsa-miR-15b-5p	FGF2
5	100	hsa-miR-15b-5p	TNRC6B
6	100	hsa-miR-15b-5p	PTPN4
7	100	hsa-miR-15b-5p	PHF19
8	100	hsa-miR-15b-5p	DESI1
9	99	hsa-miR-15b-5p	UBE2Q1
10	99	hsa-miR-15b-5p	LSM11
11	99	hsa-miR-15b-5p	NECTIN1
12	99	hsa-miR-15b-5p	GAREM1
13	99	hsa-miR-15b-5p	ANKUB1
14	99	hsa-miR-15b-5p	FBXO21
15	99	hsa-miR-15b-5p	CCNE1
16	99	hsa-miR-15b-5p	ATG14
17	99	hsa-miR-15b-5p	KIF1B
18	99	hsa-miR-15b-5p	LUZP1
19	99	hsa-miR-15b-5p	SLC13A3
20	99	hsa-miR-15b-5p	ARIH1
21	99	hsa-miR-15b-5p	MGAT4A
22	99	hsa-miR-15b-5p	EPHB2
23	99	hsa-miR-15b-5p	BTRC
24	99	hsa-miR-15b-5p	SPRYD3
25	99	hsa-miR-15b-5p	ARL2
26	99	hsa-miR-15b-5p	CASK
27	99	hsa-miR-15b-5p	NUP50

28	99	hsa-miR-15b-5p	DCLK1
29	99	hsa-miR-15b-5p	CYB561A3
30	99	hsa-miR-15b-5p	ZBTB46
31	99	hsa-miR-15b-5p	FGF7
32	98	hsa-miR-15b-5p	RECK
33	98	hsa-miR-15b-5p	PLAG1
34	98	hsa-miR-15b-5p	AXIN2
35	98	hsa-miR-15b-5p	GPR63
36	98	hsa-miR-15b-5p	SYNJ1
37	98	hsa-miR-15b-5p	ABL2
38	98	hsa-miR-15b-5p	SCN8A
39	98	hsa-miR-15b-5p	CACNA1E
40	98	hsa-miR-15b-5p	PISD
41	98	hsa-miR-15b-5p	KCNJ2
42	98	hsa-miR-15b-5p	C2orf42
43	98	hsa-miR-15b-5p	UBE2V1
44	98	hsa-miR-15b-5p	SPRED1
45	98	hsa-miR-15b-5p	SREK1
46	98	hsa-miR-15b-5p	TBL1XR1
47	98	hsa-miR-15b-5p	MTMR3
48	98	hsa-miR-15b-5p	TLK1
49	98	hsa-miR-15b-5p	SLC11A2
50	98	hsa-miR-15b-5p	MOB3B
51	98	hsa-miR-15b-5p	ZBTB44
52	98	hsa-miR-15b-5p	ANO3
53	98	hsa-miR-15b-5p	SLC9A6
54	98	hsa-miR-15b-5p	APLN
55	98	hsa-miR-15b-5p	AKT3
56	98	hsa-miR-15b-5p	IPO7
57	98	hsa-miR-15b-5p	RASGEF1B
58	98	hsa-miR-15b-5p	ATG9A
59	98	hsa-miR-15b-5p	CPEB2
60	98	hsa-miR-15b-5p	AHCYL2
61	98	hsa-miR-15b-5p	MYB
62	98	hsa-miR-15b-5p	CCND2
63	98	hsa-miR-15b-5p	KIF5C
64	98	hsa-miR-15b-5p	UBN2
65	98	hsa-miR-15b-5p	UBE4B
66	98	hsa-miR-15b-5p	ARL3
67	97	hsa-miR-15b-5p	MAMSTR
68	97	hsa-miR-15b-5p	RNF144B
69	97	hsa-miR-15b-5p	CDCA4
70	97	hsa-miR-15b-5p	KDSR
71	97	hsa-miR-15b-5p	KIF23
72	97	hsa-miR-15b-5p	TFAP2A
73	97	hsa-miR-15b-5p	PPM1E

74	97	hsa-miR-15b-5p	SHOC2
75	97	hsa-miR-15b-5p	HTR2A
76	97	hsa-miR-15b-5p	LURAP1L
77	97	hsa-miR-15b-5p	DMPK
78	97	hsa-miR-15b-5p	RAB11FIP2
79	97	hsa-miR-15b-5p	TMEM100
80	97	hsa-miR-15b-5p	RASSF8
81	97	hsa-miR-15b-5p	FBXW7
82	97	hsa-miR-15b-5p	DNAJB4
83	97	hsa-miR-15b-5p	GRM7
84	97	hsa-miR-15b-5p	WEE1
85	97	hsa-miR-15b-5p	STOX2
86	97	hsa-miR-15b-5p	CYP26B1
87	96	hsa-miR-15b-5p	N4BP1
88	96	hsa-miR-15b-5p	PLPP1
89	96	hsa-miR-15b-5p	ZNF691
90	96	hsa-miR-15b-5p	ZCCHC3
91	96	hsa-miR-15b-5p	ARMH4
92	96	hsa-miR-15b-5p	SALL4
93	96	hsa-miR-15b-5p	TBPL1
94	96	hsa-miR-15b-5p	CFAP45
95	96	hsa-miR-15b-5p	CNOT6L
96	96	hsa-miR-15b-5p	SEMA6D
97	96	hsa-miR-15b-5p	RFX3
98	96	hsa-miR-15b-5p	PCMT1
99	96	hsa-miR-15b-5p	EDA
100	96	hsa-miR-15b-5p	SEC24A
101	96	hsa-miR-15b-5p	KLHL2
102	96	hsa-miR-15b-5p	PAFAH1B1
103	96	hsa-miR-15b-5p	PTPN3
104	96	hsa-miR-15b-5p	CASR
105	96	hsa-miR-15b-5p	MYLK
106	96	hsa-miR-15b-5p	CCDC6
107	96	hsa-miR-15b-5p	NAPG
108	96	hsa-miR-15b-5p	ZNF367
109	95	hsa-miR-15b-5p	ZMAT3
110	95	hsa-miR-15b-5p	STXBP5
111	95	hsa-miR-15b-5p	UBFD1
112	95	hsa-miR-15b-5p	CHAC1
113	95	hsa-miR-15b-5p	HIPK2
114	95	hsa-miR-15b-5p	UBE4A
115	95	hsa-miR-15b-5p	CEP55
116	95	hsa-miR-15b-5p	MKX
117	95	hsa-miR-15b-5p	CBX2
118	95	hsa-miR-15b-5p	TNFSF13B
119	95	hsa-miR-15b-5p	IPPK

120	95	hsa-miR-15b-5p	VEGFA
121	95	hsa-miR-15b-5p	SLC25A37
122	95	hsa-miR-15b-5p	ZBTB34
123	95	hsa-miR-15b-5p	KIF5B
124	95	hsa-miR-15b-5p	SETD3
125	95	hsa-miR-15b-5p	PAFAH1B2
126	95	hsa-miR-15b-5p	MAP2K1
127	95	hsa-miR-15b-5p	SMURF1
128	95	hsa-miR-15b-5p	GABARAPL1
129	95	hsa-miR-15b-5p	USP25
130	95	hsa-miR-15b-5p	STXBP3
131	95	hsa-miR-15b-5p	FGFR1
132	95	hsa-miR-15b-5p	MYBL1
133	95	hsa-miR-15b-5p	GPATCH8
134	95	hsa-miR-15b-5p	CBX4
135	94	hsa-miR-15b-5p	JPH3
136	94	hsa-miR-15b-5p	USP42
137	94	hsa-miR-15b-5p	STRADB
138	94	hsa-miR-15b-5p	RNF217
139	94	hsa-miR-15b-5p	TRANK1
140	94	hsa-miR-15b-5p	LRRN3
141	94	hsa-miR-15b-5p	ZFHX4
142	94	hsa-miR-15b-5p	ZNF622
143	94	hsa-miR-15b-5p	PTPRR
144	94	hsa-miR-15b-5p	ATXN2
145	94	hsa-miR-15b-5p	OOEP
146	94	hsa-miR-15b-5p	INSR
147	94	hsa-miR-15b-5p	P3H2
148	94	hsa-miR-15b-5p	ZBTB39
149	94	hsa-miR-15b-5p	SRPRA
150	94	hsa-miR-15b-5p	PLXNA4
151	94	hsa-miR-15b-5p	WNK3
152	94	hsa-miR-15b-5p	PPP1R11
153	94	hsa-miR-15b-5p	LATS1
154	94	hsa-miR-15b-5p	DDX3X
155	94	hsa-miR-15b-5p	LRIG2
156	94	hsa-miR-15b-5p	ARHGDIA
157	94	hsa-miR-15b-5p	PPP2R1B
158	94	hsa-miR-15b-5p	DCP1A
159	94	hsa-miR-15b-5p	CHEK1
160	94	hsa-miR-15b-5p	OMG
161	94	hsa-miR-15b-5p	GALNT13
162	93	hsa-miR-15b-5p	ACTR2
163	93	hsa-miR-15b-5p	GHR
164	93	hsa-miR-15b-5p	BTAF1
165	93	hsa-miR-15b-5p	AVL9

166	93	hsa-miR-15b-5p	FBXL20
167	93	hsa-miR-15b-5p	DIXDC1
168	93	hsa-miR-15b-5p	CCND1
169	93	hsa-miR-15b-5p	2-Sep
170	93	hsa-miR-15b-5p	MFN2
171	93	hsa-miR-15b-5p	E2F3
172	93	hsa-miR-15b-5p	WNT3A
173	93	hsa-miR-15b-5p	CHD2
174	93	hsa-miR-15b-5p	ZNRF2
175	93	hsa-miR-15b-5p	SOCS6
176	93	hsa-miR-15b-5p	HSPA4L
177	93	hsa-miR-15b-5p	SEL1L3
178	93	hsa-miR-15b-5p	FOXK1
179	93	hsa-miR-15b-5p	SYT3
180	93	hsa-miR-15b-5p	HSPG2
181	93	hsa-miR-15b-5p	CD2AP
182	93	hsa-miR-15b-5p	SUCO
183	93	hsa-miR-15b-5p	MYO5A
184	93	hsa-miR-15b-5p	GATAD2A
185	93	hsa-miR-15b-5p	AMOTL1
186	93	hsa-miR-15b-5p	KANK1
187	93	hsa-miR-15b-5p	RBPJ
188	93	hsa-miR-15b-5p	SYDE2
189	93	hsa-miR-15b-5p	TMEM245
190	93	hsa-miR-15b-5p	RPS6KA3
191	93	hsa-miR-15b-5p	PLXNC1
192	93	hsa-miR-15b-5p	VPS33B
193	93	hsa-miR-15b-5p	KIF21A
194	92	hsa-miR-15b-5p	ZMYM2
195	92	hsa-miR-15b-5p	SUMO3
196	92	hsa-miR-15b-5p	CACUL1
197	92	hsa-miR-15b-5p	PIAS2
198	92	hsa-miR-15b-5p	ACVR2A
199	92	hsa-miR-15b-5p	TBP
200	92	hsa-miR-15b-5p	STK33
201	92	hsa-miR-15b-5p	SLIT2
202	92	hsa-miR-15b-5p	XPO7
203	92	hsa-miR-15b-5p	SMAD7
204	92	hsa-miR-15b-5p	YTHDC1
205	92	hsa-miR-15b-5p	SLC12A2
206	92	hsa-miR-15b-5p	POU2F1
207	92	hsa-miR-15b-5p	COP1
208	92	hsa-miR-15b-5p	ADAMTS3
209	92	hsa-miR-15b-5p	AGO4
210	92	hsa-miR-15b-5p	KCNK10
211	92	hsa-miR-15b-5p	ASH1L

212	92	hsa-miR-15b-5p	RAD23B
213	92	hsa-miR-15b-5p	SIRT4
214	92	hsa-miR-15b-5p	TMEM178B
215	92	hsa-miR-15b-5p	RNF10
216	92	hsa-miR-15b-5p	IFT74
217	92	hsa-miR-15b-5p	PTH
218	92	hsa-miR-15b-5p	G2E3
219	92	hsa-miR-15b-5p	ATXN7L2
220	92	hsa-miR-15b-5p	LITAF
221	92	hsa-miR-15b-5p	KRTAP11-1
222	92	hsa-miR-15b-5p	TRABD2B
223	92	hsa-miR-15b-5p	ZNF449
224	92	hsa-miR-15b-5p	RETREG2
225	92	hsa-miR-15b-5p	DLL1
226	92	hsa-miR-15b-5p	NHLRC2
227	92	hsa-miR-15b-5p	SLC20A2
228	92	hsa-miR-15b-5p	NAA25
229	92	hsa-miR-15b-5p	LAMP3
230	92	hsa-miR-15b-5p	TMEM183A
231	92	hsa-miR-15b-5p	CDC25A
232	92	hsa-miR-15b-5p	MEOX2
233	92	hsa-miR-15b-5p	CPEB3
234	92	hsa-miR-15b-5p	MEX3C
235	92	hsa-miR-15b-5p	PCDH17
236	91	hsa-miR-15b-5p	ATXN7L1
237	91	hsa-miR-15b-5p	SIK1
238	91	hsa-miR-15b-5p	SALL1
239	91	hsa-miR-15b-5p	CC2D1B
240	91	hsa-miR-15b-5p	TMCC1
241	91	hsa-miR-15b-5p	ZNRF3
242	91	hsa-miR-15b-5p	CLOCK
243	91	hsa-miR-15b-5p	KRTAP4-6
244	91	hsa-miR-15b-5p	CDC42SE2
245	91	hsa-miR-15b-5p	ZFHX3
246	91	hsa-miR-15b-5p	PPM1A
247	91	hsa-miR-15b-5p	CCNT1
248	91	hsa-miR-15b-5p	NOS1
249	91	hsa-miR-15b-5p	LRRK1
250	91	hsa-miR-15b-5p	RPS6KA6
251	91	hsa-miR-15b-5p	TLL1
252	91	hsa-miR-15b-5p	EZH1
253	91	hsa-miR-15b-5p	SPTLC1
254	91	hsa-miR-15b-5p	AMER1
255	91	hsa-miR-15b-5p	LARGE2
256	91	hsa-miR-15b-5p	RARB
257	91	hsa-miR-15b-5p	ZC3H13

258	91	hsa-miR-15b-5p	CSDE1
259	90	hsa-miR-15b-5p	WIPI2
260	90	hsa-miR-15b-5p	TFCP2L1
261	90	hsa-miR-15b-5p	CDK5R1
262	90	hsa-miR-15b-5p	CACNA2D1
263	90	hsa-miR-15b-5p	TGFBR3
264	90	hsa-miR-15b-5p	FAM133B
265	90	hsa-miR-15b-5p	UBQLNL
266	90	hsa-miR-15b-5p	TRIM66
267	90	hsa-miR-15b-5p	ELMSAN1
268	90	hsa-miR-15b-5p	AK4
269	90	hsa-miR-15b-5p	ABHD2
270	90	hsa-miR-15b-5p	C1orf21
271	90	hsa-miR-15b-5p	NRN1
272	90	hsa-miR-15b-5p	ADGRL1
273	90	hsa-miR-15b-5p	JARID2
274	90	hsa-miR-15b-5p	SYNRG
275	90	hsa-miR-15b-5p	USP31
276	90	hsa-miR-15b-5p	CD47
277	90	hsa-miR-15b-5p	SEMA3A
278	90	hsa-miR-15b-5p	LRIG1
279	90	hsa-miR-15b-5p	TMC7
280	90	hsa-miR-15b-5p	DENND1B
281	90	hsa-miR-15b-5p	RAB9B
282	90	hsa-miR-15b-5p	AMOT
283	90	hsa-miR-15b-5p	FERMT2
284	90	hsa-miR-15b-5p	ILDR2
285	90	hsa-miR-15b-5p	CSRNP1
286	90	hsa-miR-15b-5p	NR2C2
287	90	hsa-miR-15b-5p	ELL
288	90	hsa-miR-15b-5p	C12orf76
289	90	hsa-miR-15b-5p	BAG4

miR, microRNA; hsa : Homo Sapiens.

Table S3. Functional annotation of biological process using miR-15b-5p target genes.

ID	Term	Bonferroni Correction *p* Value
GO:0048639	positive regulation of developmental growth	0.002443
GO:0003281	ventricular septum development	0.041377
GO:0060842	arterial endothelial cell differentiation	0.015163
GO:0090287	regulation of cellular response to growth factor stimulus	0.011191
GO:0043534	blood vessel endothelial cell migration	0.026188
GO:0002040	sprouting angiogenesis	0.035021
GO:0002042	cell migration involved in sprouting angiogenesis	0.005491
GO:0033674	positive regulation of kinase activity	0.005989
GO:0045860	positive regulation of protein kinase activity	0.016370
GO:0071900	regulation of protein serine/threonine kinase activity	0.015943
GO:0071902	positive regulation of protein serine/threonine kinase activity	0.005770
GO:0016055	Wnt signaling pathway	0.000248
GO:0030111	regulation of Wnt signaling pathway	0.001420
GO:0030177	positive regulation of Wnt signaling pathway	0.000077
GO:0060070	canonical Wnt signaling pathway	0.005876
GO:0060828	regulation of canonical Wnt signaling pathway	0.002788
GO:0090263	positive regulation of canonical Wnt signaling pathway	0.000162
GO:0031331	positive regulation of cellular catabolic process	0.007719
GO:0042176	regulation of protein catabolic process	0.001474
GO:0010498	proteasomal protein catabolic process	0.001766
GO:0061136	regulation of proteasomal protein catabolic process	0.000676
GO:1903050	regulation of proteolysis involved in protein catabolic process	0.000039
GO:2000058	regulation of ubiquitin-dependent protein catabolic process	0.000164
GO:0006511	ubiquitin-dependent protein catabolic process	0.000022
GO:0043161	proteasome-mediated ubiquitin-dependent protein catabolic process	0.001944
GO:2000060	positive regulation of ubiquitin-dependent protein catabolic process	0.015135
GO:0032434	regulation of proteasomal ubiquitin-dependent protein catabolic process	0.018947
GO:0048846	axon extension involved in axon guidance	0.005747
GO:0021952	central nervous system projection neuron axonogenesis	0.015205
GO:0048588	developmental cell growth	0.004241
GO:0060284	regulation of cell development	0.000169
GO:0010720	positive regulation of cell development	0.007265
GO:0051962	positive regulation of nervous system development	0.031384
GO:0050767	regulation of neurogenesis	0.022324
GO:1990138	neuron projection extension	0.005890
GO:0050770	regulation of axonogenesis	0.011492
GO:0048675	axon extension	0.036953
GO:0050772	positive regulation of axonogenesis	0.006418

Table S4. Expressional differences based on age and gender (discovery and replication cohorts: n = 26, each).

		**miR-15b-5p**	**miR-15a-5p**	**miR-19b-3p**	**miR-27a-3p**	**miR-106b-5p**	**miR-320a-5p**	**miR-320a-3p**	**miR-451a**
Age	Ct	P = 0.547<br>r = -0.124	P = 0.487<br>r = 0.143	P = 0.510<br>r = 0.135	P = 0.957<br>r = -0.011	P = 0.527<br>r = 0.130	P = 0.903<br>r = 0.025	P = 0.998<br>r = -0.001	P = 0.355<br>r = -0.189
	ASD	P = 0.314<br>r = -0.205	P = 0.909<br>r = 0.023	P = 0.690<br>r = 0.082	P = 0.707<br>r = -0.077	P = 0.985<br>r = 0.004	P = 0.573<br>r = -0.116	P = 0.059<br>r = -0.374	P = 0.879<br>r = 0.031
Gender	Ct	P = 0.030	P = 0.978	P = 0.765	P = 0.216	P = 0.935	P = 0.461	P = 0.261	P = 0.160
	ASD	P = 0.003	P = 0.177	P = 0.461	P = 0.088	P = 0.261	P = 0.644	P = 0.144	P = 0.935

		**miR-494-5p**	**miR-494-3p**	**TGFBR3**	**MYBL1**
Age	Ct	P = 0.346<br>r = 0.193	P = 0.784<br>r = 0.057	P = 0.225<br>r = 0.246	P = 0.264<br>r = 0.227
	ASD	P = 0.825<br>r = 0.046	P = 0.499<br>r = -0.139	P = 0.909<br>r = 0.023	P = 0.580<br>r = 0.114
Gender	Ct	P = 0.338	P = 0.849	P = 0.718	P = 0.196
	ASD	P = 0.531	P = 0.216	P = 0.765	P = 0.988

ASD, Autism Spectrum disorder; miR, microRNA.

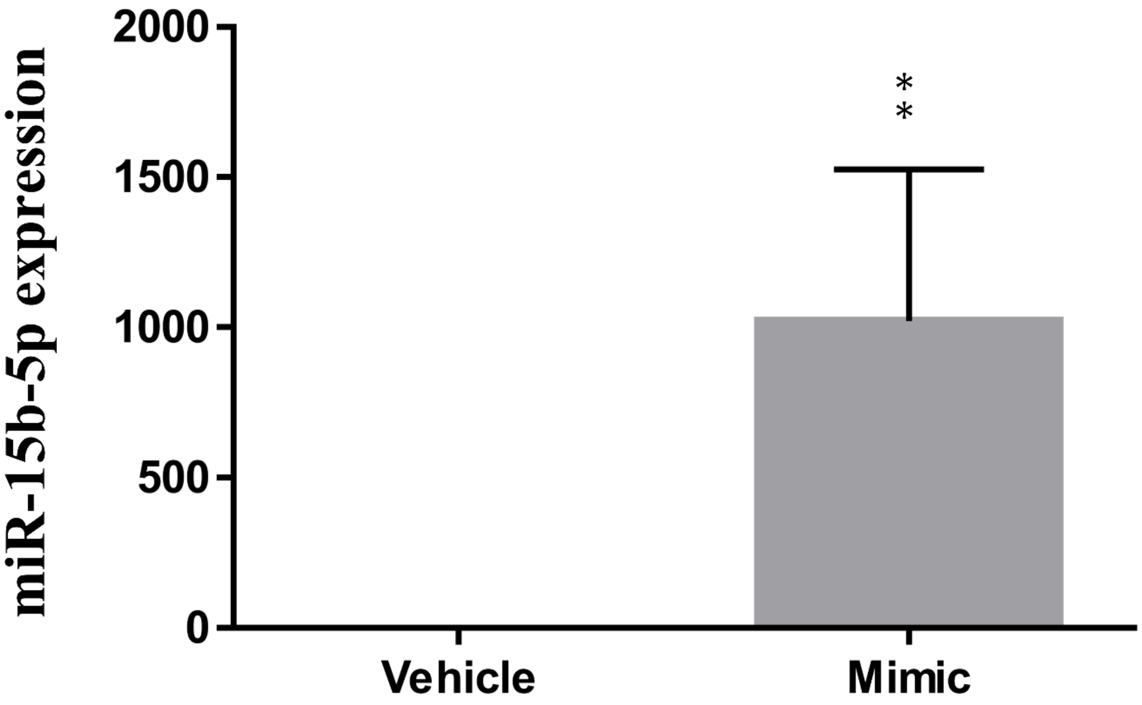

Figure S1. MiR-15b-5p expression in the miR-15b-5p oligo transfected HEK293 cells. The y-axis represents the ratio of the relative expression value of miR-15b-5p expression. miR, microRNA. * $p < 0.05$, ** $p < 0.01$.
